# Pref-1, a Gatekeeper of Adipogenesis

**DOI:** 10.3389/fendo.2013.00079

**Published:** 2013-07-03

**Authors:** Carolyn S. Hudak, Hei Sook Sul

**Affiliations:** ^1^Department of Nutritional Sciences and Toxicology, University of California, Berkeley, CA, USA

**Keywords:** Pref-1, adipogenesis, brown fat, progenitors, fibronectin, SOX9 transcription factor

## Abstract

Preadipocyte factor 1 (Pref-1, also called Dlk1/FA1) is a molecular gatekeeper of adipogenesis which acts by maintaining the preadipocyte state and preventing adipocyte differentiation. Pref-1 is made as an epidermal growth factor-like repeat containing transmembrane protein, and is cleaved by TNFα-converting enzyme (TACE) to generate a soluble form, which acts as an autocrine/paracrine factor. Pref-1 upregulates Sox9 expression by activating the ERK/MAPK pathway and the Pref-1 interaction with fibronectin is required for inhibition of adipogenesis. Pref-1 also prevents brown adipocyte differentiation and its thermogenic function. Here, we highlight the recent evidence for the role of Pref-1 in adipogenesis.

Obesity, characterized by an increase in white adipose tissue (WAT) mass, has become a major health problem on an epidemic scale and is associated with increased risk of various diseases, such as cardiovascular diseases and diabetes. Throughout the lifespan, adipose tissue not only expands through hypertrophy of existing adipocytes, but also through hyperplasia of preadipocytes differentiating into adipocytes. Various factors that affect differentiation have been identified. However, despite major advances in adipose biology, the underlying processes and mechanisms for adipose tissue development and its expansion are not fully understood. In addition, with the recent evidence of the presence of functional brown adipose tissue (BAT) in adults, implicating its potential as a therapeutic target for obesity, attention has been focused on understanding the development of BAT, which dissipates energy, in contrast to WAT that serves as the major energy storage organ.

## Adipocyte Differentiation

Adipose tissue contains heterogeneous cell types. In addition to lipid filled adipocytes, adipose tissue contains the so-called stromal vascular fraction (SVF) composed of preadipocytes, macrophages, endothelial cells, and other fibroblast-like cells. Primary preadipocytes and preadipocyte cell lines, such as 3T3-L1 and 3T3-FT442A cells, undergo differentiation in the presence of adipogenic media containing dexamethasone (DEX), isobutylmethylxanthine (IBMX), and insulin and the differentiation process has been extensively studied at the transcriptional level. Intricate coordination of various transcription factors is required for differentiation. CCAAT/enhancer binding protein β (C/EBPβ) and C/EBPδ are induced early during differentiation and activate peroxisome proliferator-activated receptor γ (PPARγ) and C/EBPα. PPARγ, is necessary and sufficient to promote adipocyte differentiation, and treatment with PPARγ agonists, thiazolidinediones, can promote differentiation *in vitro* (1). Since the discovery of PPARγ, many additional transcription factors that play a role in adipocyte differentiation have been identified and have been extensively reviewed elsewhere (2–7). Recent research has been devoted to identifying unique markers of preadipocytes or adipose progenitors. In this regard, ZFP423 has been reported to be critical for the preadipocyte commitment process (8). And, PRDM16 has been identified as a major player in the differentiation of brown adipocytes (9).

Not only does adipose tissue play a critical role in energy metabolism by being the major energy storage organ, it also secretes and responds to a variety of molecules involved in energy homeostasis, immunity, and vascular function. The role of leptin and adiponectin, two cytokines that are secreted by adipocytes and regulate energy homeostasis, have been well described elsewhere (10, 11). Inflammatory cytokines such as TNF-α and IL-6 are also secreted from adipocytes (12). TNFα may help recruit mesenchymal precursors from other organs to the adipose tissue, which may then undergo differentiation to form new adipocytes (13). We originally identified Preadipocyte factor-1 [Pref-1; also called Dlk1 (Delta-like protein-1), fetal antigen-1 (FA1)] as a preadipocyte factor that prevents adipocyte differentiation and Pref-1 expression is decreased during differentiation to allow adipogenesis (14–16). While many of the adipose related cytokines are secreted from adipocytes, Pref-1 is produced by preadipocytes and acts in an autocrine/paracrine fashion. This review highlights recent advances in the knowledge of the role of Pref-1 in the regulation of adipocyte differentiation and its signaling mechanism, as well as its effects on BAT.

## Pref-1 Structure and Signaling

Pref-1 is synthesized as a transmembrane protein that contains six EGF-like repeats at the extracellular domain (17, 18). These repeats maintain the conserved spacing to form three disulfide bonds from six cysteine residues (19). Upon proteolytic cleavage by TNF-α converting enzyme (TACE), multiple soluble forms of Pref-1 are generated and the larger 50 kDa form is biologically active. Pref-1 cleavage by TACE can be inhibited by GM6001, a broad metalloproteinase inhibitor, indicating that Pref-1 cleavage is dependent on metalloproteinase activity, and this activity is enhanced by phorbol ester treatment, showing that Protein Kinase C may regulate Pref-1 cleavage. Though DEX treatment down-regulates Pref-1 expression, regulation of Pref-1 activity may also occur at the proteolytic cleavage step (20).

We demonstrated that Pref-1 rapidly induces Sox9 expression through activation of the MEK/ERK pathway (21). Thus, Sox9 constitutive overexpression can inhibit adipocyte differentiation. Downregulation of Pref-1 during differentiation coincides with increased C/EBPβ and C/EBPδ, which occurs prior to C/EBPα and PPARγ induction. Induction of Sox9 by Pref-1 during adipocyte differentiation is at the very early stage, and Sox9 acts to maintain cells in a preadipocyte state by suppressing transcription of C/EBPβ and C/EBPδ by directly binding to their promoter regions. We also found that Pref-1 not only inhibits adipogenesis, but promotes chondrogenic commitment (21). In this regard, Sox9 has been known to function in chondrogenesis, and Sox9 ablation in mice results in the prevention of cartilage formation. We have observed that, like Sox9, ablation of Pref-1 causes malformation of skeletons in mice. Pref-1 causes inhibition of osteoblast differentiation, also via its induction of Sox9 that in turn prevents expression of an osteogenic transcription factor, Runx2. These similar *in vitro* and *in vivo* effects observed further demonstrate that physiological effects of Pref-1 are mediated via its induction of Sox9 expression.

**Figure 1:**
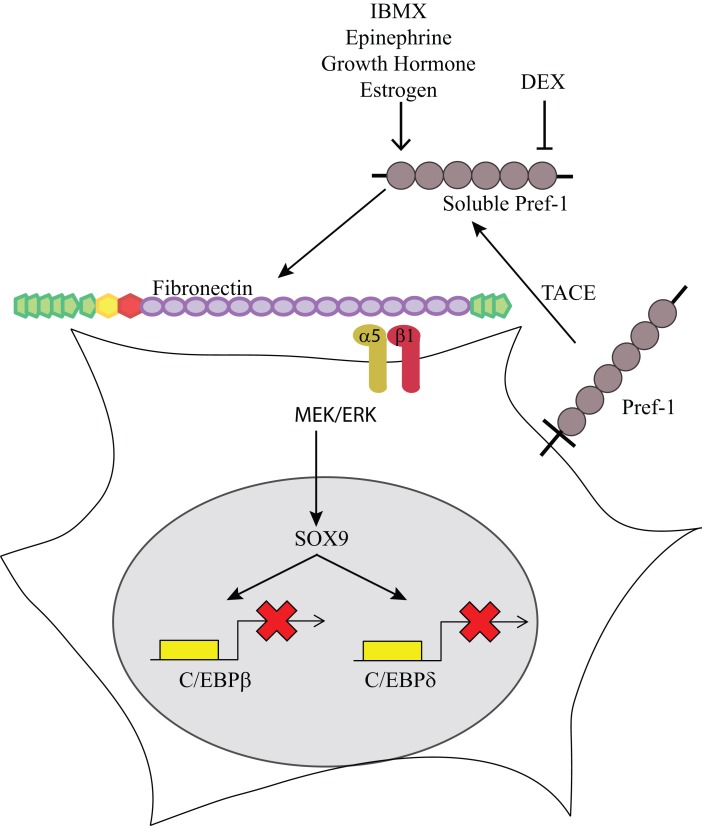
**Pref-1 inhibition of adipogenesis**. Membrane bound Pref-1 is cleaved by TACE to generate a 50 kDa soluble factor. Pref-1 interacts with fibronectin and via integrin α5β1 activates MEK/ERK, which increases Sox9, which then binds to the promoter regions of C/EBP β/δ to block adipocyte differentiation. Dexamethasone has been shown to decrease Pref-1 expression while IBMX, epinephrine, growth hormone and estrogen have been shown to increase Pref-1 expression.

The mechanistic details of the Pref-1 signaling pathway have been recently uncovered. Although it has been proposed that Pref-1 may function in the Notch signaling pathway due to the presence of EGF repeats (22, 23), Pref-1 lacks the Delta:Serrate:Lin-12 (DSL) domain that is required to mediate activation of Notch. Furthermore, we found that there is no direct interaction between Pref-1 and Notch, nor were there any changes in the expression of a downstream target of Notch, Hes-1, by Pref-1. Though the putative Pref-1 receptor is yet to be identified, we found that Pref-1 interacts with fibronectin (24). The Pref-1 interaction with the C-terminal domain of fibronectin is necessary for the Pref-1 inhibition of adipocyte differentiation. Several growth factors are known to bind various regions of fibronectin for modulation of growth factor function. Furthermore, fibronectin was shown previously to prevent adipocyte differentiation (25, 26). We found that knockdown of fibronectin or addition of Pref-1 interacting domains of fibronectin prevent the Pref-1 mediated activation of MEK/ERK and Sox9 induction, resulting in enhancement of adipocyte differentiation. Furthermore, knockdown of the α5 of the major integrin in preadipocytes, α5β1, or treatment with RGD peptide which blocks the fibronectin interaction, prevent the Pref-1 inhibitory action on adipocyte differentiation. Pref-1-fibronectin-integrin interaction activates the FAK/Src complex and Rho-like GTPases, which can then activate the ERK/MAPK pathway (27). Overall, Pref-1 directly binds fibronectin and activates integrin signaling through ERK/MAPK to inhibit adipocyte differentiation (24) (Figure 1).

## Role of Pref-1 during Adipogenesis

As described above, Pref-1 was originally identified as an inhibitor of adipocyte differentiation and its effect on WAT development has been well documented *in vitro* and *in vivo*. Overexpression of Pref-1 in 3T3-L1 preadipocytes drastically lowers the degree of adipocyte differentiation, and knocking down Pref-1 greatly increases differentiation (14, 28). Furthermore, we have generated Pref-1 null mice as well as transgenic mice overexpressing Pref-1 (29–31). Pref-1 null mice have increased adiposity and also higher mRNA levels of adipocyte markers. On the other hand, mice overexpressing Pref-1 have decreased adipose mass and decreased adipocyte marker expression. As observed in lipodystrophy models, these mice also have hypertriglyceridemia, decreased glucose tolerance, and lower insulin sensitivity (31). We can conclude that Pref-1 clearly affects adipogenesis.

The role of Pref-1 in BAT has not been well explored. As in WAT, however, it is known that Pref-1 is downregulated during BAT development, being present at a very high level in fetuses but decreasing to an undetectable level several weeks after birth (32). Though BAT appeared to be normal prior to birth, Pref-1 ablated mice show altered BAT morphology after birth, with potential thermogenic over-activation, indicated by the induction of thermogenic markers and enhanced lipid mobilization. Conversely, in lipodystrophy mouse models, Pref-1 is expressed at a higher level in BAT, which is associated with loss of thermogenesis and increased lipid accumulation (33). An indirect mechanism of this effect may be through the DEX-mediated induction of C/EPBδ, as was recently demonstrated by Armengol *et al*. (32). Taken together, these results demonstrate that Pref-1 plays an important role in the regulation of both WAT and BAT differentiation.

Dexamethasone suppresses Pref-1 expression in preadipocytes allowing for the eventual induction in PPARγ expression and differentiation of preadipocytes into adipocytes. Though the adipocyte differentiation process has been well studied, little is known about the transition steps from embryonic stem cells (ESC) or mesenchymal stem cells (MSC) to preadipocytes (34). A potential role of Pref-1 in the commitment stage of adipogenesis has been revealed via the use of MSCs. These pluripotent MSCs can differentiate into adipocytes upon treatment with IBMX and DEX. It has recently been reported that IBMX increases Pref-1 expression initially (35), followed by a DEX-mediated decrease in Pref-1 expression, showing a transient rise in the Pref-1 level in MSCs at the commitment stage prior to adipocyte differentiation. Similarly, during differentiation of human fetal MSCs into adipocytes, the Pref-1 expression level rose early in the differentiation process but decreased in the later stages. Furthermore, epinephrine treatment in mouse ESC was reported to induce Pref-1 expression and cell proliferation. Interestingly, the Pref-1 level was increased at the commitment stage, indicating that epinephrine may induce cells to become committed to the adipocyte lineage. This effect could be reversed by treatment with antagonists of the Neuropeptide Y (NPY) receptor (36). These observations together suggest that commitment to the adipocyte lineage involves Pref-1 induction (37), and Pref-1 may be used as a marker for precursors of adipogenesis.

The identity of adipose precursors remains an active area of research. Lineage tracing has often been used to understand the developmental origin of various cell types (38–40). By using the PPARγ locus, Tang et al. (41) first showed that adipose progenitors express PPARγ, and they reside near the vasculature. However, PPARγ is expressed not only in preadipocytes but also in differentiated adipocytes, which makes it tricky to discern adipose precursors. Regardless, other recent reports also indicate that adipocytes may come from endothelial cells or pericytes (8, 42, 43). Adipocyte precursor cells were also identified in the adipose stroma using a combination of fluorescence-activated cell sorting. These cells were found to have a unique cell surface signature, including the presence of CD34, Sca-1, and CD24 (44). And, we recently employed Pref-1 labeling to identify and characterize adipose precursors throughout development, as well as during diet induced expansion of WAT (Hudak and Sul, unpublished results).

In humans, Pref-1 has been recently shown to be clinically relevant and is associated with extreme early onset obesity. In trio studies of obese children and their biological parents, the association of Pref-1 with obesity became apparent only when considering imprinting of the gene, and supported the existence of polar overdominance in humans (45). Furthermore, in comparing Pref-1 expression levels in metabolically healthy obese (MHO) and metabolically unhealthy obese (MUO) individuals, MHO patients had lower levels of Pref-1 in their adipose tissue (46). These results indicated that metabolically healthy patients have increased preadipocyte differentiation which was also associated with a more favorable inflammatory profile. Therefore, MHO patients may have an increased number of smaller adipocytes, rather than increased adipocyte size, implying that increased adipocyte size may be an important process underlying metabolic disease in obesity.

## Regulation of Pref-1 Expression during Adipogenesis

Pref-1 is part of the imprinted gene cluster, Pref-1-Dio3 (47, 48). Ten imprinted genes have been identified in this cluster. There are three paternally expressed genes, Pref-1, Paternally Expressed Gene 11 [Peg11, also called Retrotransposon-like 1(Rtl1)] and type 3 deiodinase (Dio3), and seven maternally expressed genes coding for non-coding RNAs (48, 49). Interestingly, Pref-1 is located in the same imprinted region as *Dio3* (50), an inhibitor of T3, and changes in *Dio3* expression levels during differentiation parallel that of Pref-1. Thyroid hormone is critical for BAT function and thermogenesis (51, 52), and T3 can stimulate brown adipocyte differentiation (53). Accordingly, DEX can suppress expression of both Dio3 and Pref-1 in cultured preadipocytes. In this regard, high levels of Dio3 have been detected in differentiating primary brown preadipocytes. Together, the coordinated expression of Pref-1 and Dio3 further implicate the important role they may play during brown adipocyte differentiation (54). Not only is DNA methylation important in the regulation of Pref-1 expression, but modification of histones also plays a role in expression of these imprinted genes. With regard to Pref-1 repression during adipogenesis, a recent study revealed that a decreased level of H3 K4 tri-methylation (55) leads to de-repression of Pref-1, and the Pref-1 locus exhibits lower levels of active chromatin makers, such as H3 K4 tri-methylation as well as H3 and H4 acetylation in 10T1/2 cells compared to committed 3T3-L1 preadipocytes. Furthermore, the Pref-1 gene showed a predominant enrichment of H3 K27 tri-methylation, demonstrating that these repressive marks play a role in silencing Pref-1 gene expression in 10T1/2 cells (56, 57).

Regulation of adipogenesis by certain growth factors has been reported to be through modulating Pref-1 levels. Thus, growth hormone has been previously reported to maintain high levels of Pref-1 in 3T3-L1 cells to prevent differentiation of preadipocytes (58, 59). Furthermore, growth hormone stimulates production of Insulin-like growth factor-1 (IGF-1), which is reduced by Pref-1 treatment, showing the ability of Pref-1 to regulate growth hormone levels (60). It has also been suggested that IGF-1 may be able to bypass the inhibition of Pref-1 on adipocyte differentiation of 3T3-L1 cells (61). Interestingly, in human patients with anorexia nervosa, there was an increased level of Pref-1, which correlated to an increased number of undifferentiated cells with a corresponding decrease in BAT mass (62). Furthermore, these patients have low gonadal steroids and treating adolescent female anorexia nervosa with estradiol caused a significant decrease in Pref-1 levels (63). In postmenopausal women, estrogen deficiency leads to increased serum levels of Pref-1, which is associated with bone loss, and could be normalized with estrogen replacement therapy (64). Overall, there seems to be evidence of estrogen regulation of Pref-1 expression, which plays a role in regulating homeostasis of adipocyte as well as osteocyte abundance.

## Conclusion

Obesity is characterized by increased adipose tissue through both hypertrophy and hyperplasia, and much interest has been garnered to understand the regulation of this process. In this regard, Pref-1 has been used as a preadipocyte marker, and is notable in its ability to prevent adipocyte differentiation in an autocrine/paracrine manner. Pref-1 interacts with fibronectin and activates MEK/ERK to induce Sox9 expression, which blocks adipocyte differentiation by binding to the promoter regions of C/EBPβ and C/EBPδ and preventing activation of C/EBPα and PPARγ. Several hormones and other cytokines have been suggested to either regulate or be regulated by Pref-1, further suggesting the critical role of Pref-1 in the maintenance of the preadipocyte state. Pref-1 expression transiently increases early during differentiation of MSCs. Pref-1 may be a valuable tool as a marker of precursor cells and we are employing Pref-1 to label adipose precursors for lineage tracing and investigation of adipose tissue development. Additionally, given that imprinted genes function in fetal development and growth and with its effects on chondrogenesis and osteogenesis, Pref-1 most likely functions beyond the control of adipogenesis and mesenchymal cell fate. Furthermore, Pref-1 is associated with early onset obesity in humans. Uncovering the Pref-1-fibronectin interaction has begun to shed light on the molecular mechanism underlying the function of Pref-1 and Pref-1 signaling in adipogenesis.

## Conflict of Interest Statement

The authors declare that the research was conducted in the absence of any commercial or financial relationships that could be construed as a potential conflict of interest.
